# Stroke incidence in heart failure and atrial fibrillation: a population-based retrospective cohort study

**DOI:** 10.3399/BJGP.2024.0470

**Published:** 2025-03-11

**Authors:** Nicholas R Jones, Margaret Smith, Sarah Lay-Flurrie, Yaling Yang, Richard Hobbs, Clare J Taylor

**Affiliations:** Nuffield Department of Primary Care Health Sciences, University of Oxford, Oxford.; Nuffield Department of Primary Care Health Sciences, University of Oxford, Oxford; NIHR Oxford Biomedical Research Centre, Oxford.; Nuffield Department of Primary Care Health Sciences, University of Oxford, Oxford.; Nuffield Department of Primary Care Health Sciences, University of Oxford, Oxford.; Nuffield Department of Primary Care Health Sciences, University of Oxford, Oxford.; Nuffield Department of Primary Care Health Sciences, University of Oxford, Oxford; Department of Applied Health Sciences, University of Birmingham, Birmingham.

**Keywords:** atrial fibrillation, heart failure, mortality, primary care, risk prediction, stroke

## Abstract

**Background:**

Heart failure (HF) is a risk factor for stroke among people with atrial fibrillation (AF). Prognosis following an HF diagnosis is often poor, but this is not accounted for in existing stroke risk scores.

**Aim:**

To examine stroke incidence in people with HF and AF compared with AF alone, considering the competing risk of death.

**Design and setting:**

A population-based retrospective cohort study in English primary care, linked to secondary care Hospital Episode Statistics data.

**Method:**

In total, 2 381 941 people aged ≥45 years were identified in the Clinical Practice Research Datalink from 2000 to 2018. HF and AF were included as time-varying covariates; 69 575 had HF and AF, 141 562 had AF alone, and 91 852 had HF alone. Hazard ratios (HRs) for first stroke are reported using the Cox model and the Fine–Gray model.

**Results:**

Over median follow-up of 6.62 years, 93 665 people (3.9%) had a first stroke and 314 042 (13.2%) died. Over half (51.3%) of those with HF, with or without AF, died. In the fully adjusted Cox model, relative stroke risk was highest among people with AF alone (HR 2.43, 95% confidence interval [CI] = 2.38 to 2.48), followed by HF and AF (HR 2.20, 95% CI = 2.14 to 2.26). The cumulative incidence function of stroke was also highest among those with AF only once accounting for the competing risk of all-cause mortality. In a Fine–Gray model, the relative risk of stroke was similar for people with AF alone (HR 2.38, 95% CI = 2.33 to 2.43), but there was significant attenuation among those with HF and AF (HR 1.48, 95% CI = 1.44 to 1.53).

**Conclusion:**

HF is an aetiological risk factor for stroke, yet its prognostic significance is reduced by the high incidence of death. Use of the CHA_2_DS_2_-VASc score may overestimate stroke incidence in some people with HF, particularly those with a poor prognosis.

## Introduction

Atrial fibrillation (AF) is associated with a near five-fold increased risk of stroke.^1^ International guidelines recommend that clinicians should estimate stroke risk for all individuals with AF using a validated prediction tool, such as CHA_2_DS_2_-VASc.^2^^,^^3^ People with a CHA_2_DS_2_-VASc score ≥2 are considered high risk and should be offered an anticoagulant for stroke prevention, unless there is a contraindication. Heart failure (HF) is also associated with increased stroke risk and scores one point in CHA_2_DS_2_-VASc.^4^ Patients with HF are typically older (median age at diagnosis is 76 years), meaning most people who have AF and HF are considered at high risk of stroke.^5^

The prognosis for people with HF is often poor, with mean 5-year mortality close to 50%.^6^ This is important when considering stroke incidence and the benefits of anticoagulation, because people with a short life expectancy will have less time when they are exposed to the risk of stroke. Statistically, death is considered a competing risk, defined as *‘an event whose occurrence precludes the occurrence of the primary event of interest’.*^7^

When CHA_2_DS_2_-VASc was developed, this competing risk of death was not accounted for.^8^ Traditional survival analysis approaches that do not consider competing risks typically overestimate the prognostic importance of a variable. For example, the Kaplan–Meier (KM) method assumes that censoring is non-informative and that, if follow-up were to have continued for long enough, the primary outcome would have occurred in all individuals.^9^ If the competing risk is death, this cannot be true. When competing risks are accounted for in a cumulative incidence function (CIF), each individual can only have one event, meaning that the CIF is equal to the composite of the competing risk outcomes.^7^

This study aimed to determine the stroke incidence among patients with HF and AF compared with people with AF alone, incorporating a competing risks analysis to explore the importance of HF when assessing stroke risk in people with AF.

## Method

A retrospective cohort study using primary care data from the Clinical Practice Research Datalink (CPRD) was conducted between January 2000 and December 2018. CPRD data were linked to inpatient Hospital Episode Statistics (HES) and Office for National Statistics (ONS) data. An Independent Scientific Advisory Committee approved the CPRD access (protocol: 19_125).

**Table:** How this fits in

Heart failure (HF) is a stroke risk predictor in the CHA_2_DS_2_-VASc score for people with atrial fibrillation (AF), yet the patient’s overall prognosis is not considered when making this estimation. This study found that people with HF and AF were at an increased risk of stroke, but a much greater risk of death, with 61.0% of people with both conditions dying. After accounting for this competing risk, and adjusting for comorbidities, the relative risk of stroke was higher in people with AF alone compared with people with HF and AF. HF may be of limited value in predicting stroke risk in people with AF whose overall prognosis is poor. However, many people with HF and AF will still benefit from an anticoagulant to prevent stroke and younger patients may have most to gain.

### Study population

All patients in CPRD GOLD aged ≥45 years and registered at a participating ‘up-to-standard practice’ for at least 12 months were included^10^ and eligible for data linkage, which limited the study to England. Patients with a prior history of stroke were excluded. Read and SNOMED CT codes were used to identify variables in CPRD, and *International Classification of Diseases, Tenth Revision* codes in HES (Supplementary Appendix S1).

The index date when patients entered the cohort was defined as the latest of the following: 1 January 2000; date of 45^th^ birthday; patient registration date plus 12 months; or practice up-to-standard date plus 12 months. Patients exited the cohort on the earliest of the following dates: 31 December 2018; transfer out of CPRD-registered practice; date of death; last date of practice data collection; or the last date of available linked data.

### Exposure

Participants were categorised as having HF and AF, HF alone, AF alone, or neither condition based on the earliest diagnosis in either their primary or secondary care electronic health record. This included participants who had either condition prior to the study and all incident cases during follow-up. HF/AF category was included as a time-varying covariate.^11^

### Primary outcome

This was incidence of stroke, subcategorised as ischaemic, haemorrhagic, or type unspecified, as recorded in either the primary or secondary care patient record (Supplementary Appendix S1).

### Covariates

Covariates included all remaining elements of the CHA_2_DS_2_-VASc score (hypertension, age, sex, diabetes, thromboembolic disease excluding stroke, and vascular disease), ethnicity, and smoking status, defined by the presence of a code in the patient’s electronic health record at any time before the index date, or before an incident diagnosis of HF or AF. Data were also collected on body mass index, frailty score, presence of chronic kidney disease, or migraine and anticoagulation treatment, defined as any prescription for either warfarin or a direct oral anticoagulant in the first 90 days after a new diagnosis of AF or HF.

### Statistical analysis

Initial analyses included a descriptive summary of the baseline characteristics of the population. The crude and age-stratified incidence rates of stroke by presence of HF and/or AF per 1000 person–years at risk and time at risk are reported.^12^^,^^13^

A Cox proportional hazards model and a Fine–Gray model were conducted to estimate the hazard of a first stroke between exposure groups, with the latter accounting for the competing risk of all-cause mortality.^14^ For both models, unadjusted hazard ratio (HR), adjusted for age and sex, and adjusted for the pre-specified cardiovascular risk factors, as listed above, are reported. Cause-specific HRs from the Cox model are useful for considering an aetiological link between HF and AF with stroke. The HRs from the Fine–Gray model are more appropriate for estimating to what extent HF and AF impact on the absolute risk of stroke over time, such as when estimating prognosis.^15^

Subgroup and post-estimation analyses based on anticoagulation, by the age ranges used in the CHA_2_DS_2_-VASc score and by categories of CHA_2_DS_2_-VASc score, were undertaken.

A landmark analysis starting from the index date then at each year of follow-up, to year 15, was conducted. The KM failure function and CIF were calculated to estimate the cumulative probability of stroke at 3-month follow-up and at 1-, 2-, 5-, and 10-year follow-up from each landmark year, and then used inverse variance weighting to calculate the mean cumulative probability.

As a sensitivity analysis, both the Cox and Fine–Gray models were repeated in a landmark analysis, with individuals’ exposure status defined at the index date and at 5-year follow-up, without allowing for subsequent change in disease status.^16^ A further sensitivity analysis was conducted including fatal stroke events reported in ONS within the study stroke outcomes, even among patients without a recorded stroke in either CPRD or HES, as concordance between coding across datasets has been shown to be poor.^17^

There were substantial missing data for smoking status and ethnicity, which was unlikely to be missing at random.^18^ Multiple imputation was therefore considered inappropriate, so a new ‘missing’ variable category was created for the main analysis. A complete case analysis for comparison was also carried out. Recording of diagnostic codes was assumed to be complete. The absence of a prescription code was interpreted as no treatment prescribed.

All analyses were conducted in Stata (version 14).

## Results

### Baseline characteristics

The cohort included 2 381 941 patients with a median follow-up time of 6.62 years (range 0–18.9). At the index date, there were 31 079 people with HF alone, 40 582 with AF alone, and 16 213 with both AF and HF (Table 1). During the study period, 91 852 people had HF alone, 141 562 had AF alone, and 69 575 had both AF and HF (Table 2).

**Table 1.** Baseline characteristics by presence of heart failure and/or atrial fibrillation at index date

**Characteristic, *n* (%)^a^**	**Neither HF nor AF, *n* = 2 294 067**	**HF alone, *n* = 31 079**	**AF alone, *n* = 40 582**	**HF and AF, *n* = 16 213**	**Total, *N* = 2 381 941**
**Age at entry into study, years, mean (SD)**	56.2 (12.2)	75.9 (12.2)	72.6 (12.9)	78.2 (10.9)	57.0 (12.7)

**Patient sex**					
Male	1 109 577 (48.4)	14 163 (45.6)	21 585 (53.2)	7675 (47.3)	1 153 000 (48.4)
Female	1 184 490 (51.6)	16 916 (54.4)	18 997 (46.8)	8538 (52.7)	1 228 941 (51.6)

**Ethnicity**					
White	1 615 304 (70.4)	25 687 (82.7)	36 117 (89.0)	14 289 (88.1)	1 691 397 (71.0)
Non-White	100 999 (4.4)	1357 (4.4)	987 (2.4)	375 (2.3)	103 718 (4.4)
Unknown	577 764 (25.2)	4035 (13.0)	3478 (8.6)	1549 (9.6)	586 826 (24.6)

**Smoking status**					
Non-smoker	1 063 899 (46.4)	13 386 (43.1)	19 305 (47.6)	7348 (45.3)	1 103 938 (46.3)
Current smoker	466 287 (20.3)	4157 (13.4)	4444 (11.0)	1560 (9.6)	476 448 (20.0)
Ex-smoker	451 902 (19.7)	8926 (28.7)	12 038 (29.7)	5022 (31.0)	477 888 (20.1)
Missing	311 979 (13.6)	4610 (14.8)	4795 (11.8)	2283 (14.1)	323 667 (13.6)

**Body mass index, kg/m^2^, mean (SD)**	26.6 (4.8)	27.7 (5.2)	26.8 (4.9)	26.9 (5.3)	26.6 (4.8)

**Chronic kidney disease^b^**	22 035 (1.0)	1956 (6.3)	2351 (5.8)	1857 (11.5)	28 199 (1.2)

**Diabetes**	112 425 (4.9)	5585 (18.0)	4578 (11.3)	2949 (18.2)	125 537 (5.3)

**Hypertension**	427 629 (18.6)	14 190 (45.7)	18 272 (45.0)	7871 (48.5)	467 962 (19.6)

**Liver disease**	9699 (0.4)	200 (0.6)	238 (0.6)	81 (0.5)	10 218 (0.4)

**Migraine**	128 596 (5.6)	1007 (3.2)	1599 (3.9)	466 (2.9)	131 668 (5.5)

**Thromboembolism**	13 806 (0.6)	999 (3.2)	1047 (2.6)	729 (4.5)	16 581 (0.7)

**Vascular disease, including MI and PAD^c^**	59 571 (2.6)	9177 (29.5)	4962 (12.2)	3882 (23.9)	77 592 (3.3)

**Frailty category**					
Fit	2 131 093 (92.9)	12 528 (40.3)	23 030 (56.7)	4417 (27.2)	2 171 068 (91.1)
Mild frailty	150 405 (6.6)	14 406 (46.4)	14 714 (36.3)	8233 (50.8)	187 758 (7.9)
Moderate frailty	11 943 (0.5)	3726 (12.0)	2572 (6.3)	3077 (19.0)	21 318 (0.9)
Severe frailty	626 (0.03)	419 (1.3)	266 (0.7)	486 (3.0)	1797 (0.1)

**Anticoagulant**					
None	2 284 819 (99.6)	29 448 (94.8)	29 667 (73.1)	10 205 (62.9)	2 354 139 (98.8)
VKA	8912 (0.4)	1602 (5.2)	10 326 (25.4)	5727 (35.3)	26 567 (1.1)
DOAC	336 (0.02)	29 (0.1)	589 (1.5)	281 (1.7)	1235 (0.1)

**Antiplatelet**					
None	2 145 322 (93.5)	16 662 (53.6)	24 952 (61.5)	9257 (57.1)	2 196 193 (92.2)
Dual	3289 (0.1)	600 (1.9)	312 (0.8)	209 (1.3)	4410 (0.2)
High dose	5979 (0.3)	560 (1.8)	864 (2.1)	334 (2.1)	7737 (0.3)
Standard dose	139 365 (6.1)	13 256 (42.7)	14 453 (35.6)	6413 (39.6)	173 487 (7.3)
Other	112 (0.005)	1 (0.003)	1 (0.002)	0 (0.0)	114 (0.005)

a *Unless otherwise stated.*

b *Chronic kidney disease included as coded in the patient’s electronic health record.*

c *Vascular disease included codes for ischaemic heart disease, myocardial infarction, aortic plaque, or peripheral arterial disease. AF = atrial fibrillation. DOAC = direct oral anticoagulant. HF = heart failure. MI = myocardial infarction. PAD = peripheral arterial disease. SD = standard deviation. VKA = vitamin K antagonist.*

**Table 2.** Number of deaths or first strokes in relation to presence of heart failure and/or atrial fibrillation

**Category**	**Neither HF nor AF**	**HF alone**	**AF alone**	**HF and AF**	**Total**
**Number at risk^a^**	2 294 027	91 852	141 562	69 575	2 381 941

**Total number of deaths (% of population at risk)**	191 680 (8.4)	40 379 (44.0)	39 556 (27.9)	42 427 (61.0)	314 042 (13.2)

**Deaths with stroke listed as a primary or secondary cause (% of total deaths)**	13 594 (7.1)	2271 (5.6)	5377 (13.6)	3827 (9.0)	25 069 (8.0)

**Total number of strokes (% of population at risk)**	66 002 (2.9)	5413 (5.9)	15 277 (10.8)	6973 (10.0)	93 665 (3.9)

**Proportion of strokes by type (% of total number of strokes for column subgroup)^b^**					
Ischaemic stroke	23 029 (34.9)	2125 (39.3)	7672 (50.2)	3373 (48.4)	36 199 (38.6)
Haemorrhagic stroke	10 368 (15.7)	671 (12.4)	2029 (13.3)	988 (14.2)	14 056 (15.0)
Stroke uncertain subtype	32 605 (49.4)	2617 (48.3)	5576 (36.5)	2612 (37.5)	43 410 (46.3)

a *Note the total number at risk exceeds the number of participants in the study because participants could move between groups over the course of follow-up.*

b *Any stroke includes intracranial bleeds as well as ischaemic stroke events. AF = atrial fibrillation. HF = heart failure.*

Compared with people with AF alone, people with HF and AF were on average older, had a higher prevalence of hypertension, diabetes, chronic kidney disease, and moderate-to-severe frailty, and a greater proportion were women (Table 1).

### Stroke incidence

During follow-up, 93 665 (3.9%) people had a first stroke, including 12 386/161 426 (7.7%) of those with HF (Table 2). The median time to first stroke was 5.85 years (interquartile range 2.64–9.79) (data not shown). The crude incidence rate of stroke per 1000 person–years at risk was 37.3 among people with HF and AF (95% confidence interval [CI] = 36.5 to 38.2), compared with 29.9 (95% CI = 29.4 to 30.3) among people with AF alone (Supplementary Table S1).

**Figure 1.**
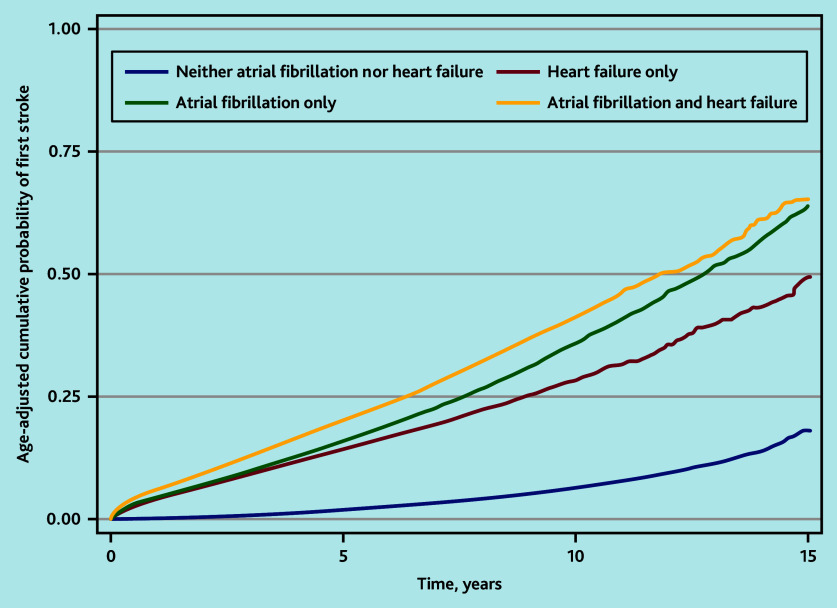
Kaplan–Meier failure function for the age-adjusted cumulative probability of first stroke over time, by presence of heart failure and/or atrial fibrillation.

In the age-adjusted KM (Figure 1) and unadjusted Cox model, the HR of stroke was highest in people with HF and AF (HR 8.93, 95% CI = 8.71 to 9.16), followed by people with AF alone (HR 7.12, 95% CI = 7.00 to 7.25) (Table 3). However, in the fully adjusted Cox model, the relative rate of stroke was highest in people with AF alone (HR 2.43, 95% CI = 2.38 to 2.48), followed by people with HF and AF (HR 2.20, 95% CI = 2.14 to 2.26).

**Table 3.** Hazard ratios of first stroke for people with heart failure and/or atrial fibrillation, comparing a Cox proportional hazards model with a Fine–Gray competing risks model

**Category**	**Patients with an incident stroke, *n***	**Unadjusted**		**Age and sex adjusted**		**Full model^a^**	
		**Cox model, HR (95% CI)**	**Fine–Gray model, subdistribution, HR (95% CI)**	**Cox model, HR (95% CI)**	**Fine–Gray model, subdistribution, HR (95% CI)**	**Cox model, HR (95% CI)**	**Fine–Gray model, subdistribution, HR (95% CI)**
**Neither HF nor AF**	66 002	Reference		Reference		Reference	
**HF alone**	5413	4.77 (4.64 to 4.90)	2.86 (2.78 to 2.94)	1.59 (1.54 to 1.63)	1.15 (1.11 to 1.18)	1.40 (1.36 to 1.45)	1.04 (1.01 to 1.08)
**AF alone**	15 277	7.12 (7.00 to 7.25)	5.87 (5.76 to 5.98)	2.60 (2.55 to 2.65)	2.65 (2.60 to 2.71)	2.43 (2.38 to 2.48)	2.38 (2.33 to 2.43)
**HF and AF**	6973	8.93 (8.71 to 9.16)	4.79 (4.66 to 4.91)	2.47 (2.41 to 2.67)	1.70 (1.66 to 1.75)	2.20 (2.14 to 2.26)	1.48 (1.44 to 1.53)

a *Adjusted for age, sex, hypertension, diabetes, history of thromboembolic or vascular disease, smoking, and ethnicity. AF = atrial fibrillation. HF = heart failure. HR = hazard ratio.*

A greater proportion of stroke events were classified as ischaemic in people with AF, with or without HF, whereas people without AF had a greater proportion of unspecified strokes (Table 2). Median and mean length of hospital admission related to stroke did not differ significantly between groups (Supplementary Table S2).

### Competing risks analysis

There were 314 042 deaths, including 25 069 where stroke was the primary or secondary cause (Table 2). More than half of people with HF, with or without AF, died during follow-up (*n* = 82 806 deaths, 51.3%), compared with 27.9% of people with AF alone.

In the fully adjusted Fine–Gray model, the HR for first stroke remained more than double among people with AF alone compared with the population with neither HF nor AF (subdistribution HR 2.38, 95% CI = 2.33 to 2.43), but there was significant attenuation of the HRs among people with HF and AF (HR 1.48, 95% CI = 1.44 to 1.53) (Table 3).

**Figure 2.**
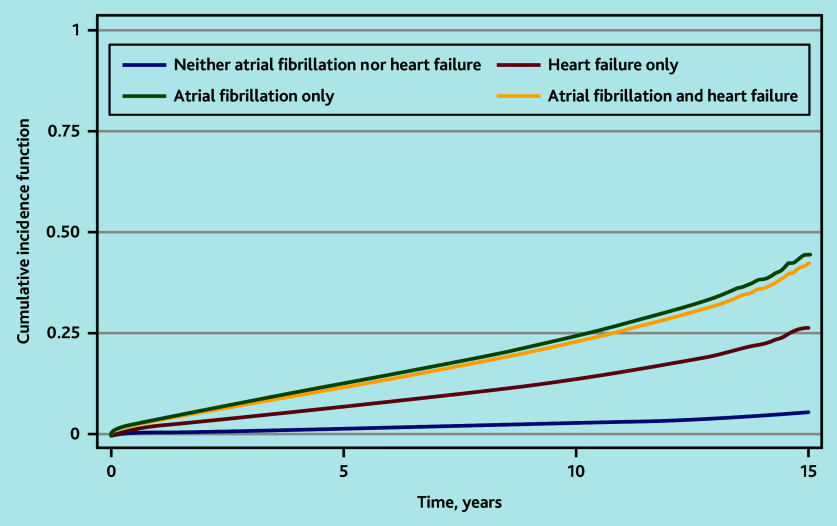
Cumulative incidence function of first stroke by presence of heart failure and/or atrial fibrillation.

Accounting for the competing risk of all-cause mortality, the cumulative incidence of stroke was similar among people with HF and AF or AF alone (Figure 2). The cumulative probability of stroke at 3-month and 1-, 2-, 5-, and 10-year follow-up are shown in Table 4. The KM failure function overestimated the cumulative probability of stroke compared with the CIF.

**Table 4.** Incidence of first stroke at 3-month and 1-, 2-, 5-, and 10-year follow-up in people with heart failure and/or atrial fibrillation, estimated using the cumulative probability via *sts list* and the cumulative incidence function via *stpm2*

**Analysis**	**Follow-up period**				

	**3 months, incidence (95% CI)**	**1 year, incidence (95% CI)**	**2 years, incidence (95% CI)**	**5 years, incidence (95% CI)**	**10 years, incidence (95% CI)**
**Neither HF nor AF**					
Cumulative incidence function	0.12 (0.11 to 0.13)	0.50 (0.48 to 0.52)	1.02 (1.00 to 1.05)	2.62 (2.58 to 2.67)	5.17 (5.12 to 5.22)
Kaplan–Meier failure function	0.12 (0.11 to 0.13)	0.50 (0.49 to 0.52)	1.04 (1.02 to 1.07)	2.72 (2.68 to 2.76)	5.56 (5.50 to 5.62)

**HF alone**					
Cumulative incidence function	0.47 (0.37 to 0.58)	1.68 (1.48 to 1.90)	3.19 (2.90 to 3.50)	6.89 (6.44 to 7.34)	11.1 (10.5 to 11.6)
Kaplan–Meier failure function	0.47 (0.38 to 0.60)	1.77 (1.56 to 2.00)	3.51 (3.19 to 3.86)	8.62 (8.04 to 9.20)	17.2 (16.2 to 18.1)

**AF alone**					
Cumulative incidence function	0.55 (0.47 to 0.64)	2.06 (1.90 to 2.23)	3.95 (3.71 to 4.19)	8.92 (8.55 to 9.32)	15.2 (14.7 to 15.7)
Kaplan–Meier failure function	0.55 (0.48 to 0.65)	2.11 (1.95 to 2.29)	4.15 (3.91 to 4.41)	10.1 (9.68 to 10.5)	19.4 (18.8 to 20.2)

**HF and AF**					
Cumulative incidence function	0.71 (0.57 to 0.88)	2.47 (2.18 to 2.78)	4.54 (4.13 to 4.98)	9.12 (8.51 to 9.76)	13.4 (12.7 to 14.2)
Kaplan–Meier failure function	0.73 (0.58 to 0.91)	2.64 (2.34 to 2.99)	5.16 (4.70 to 5.67)	12.3 (11.4 to 13.1)	23.0 (21.4 to 24.6)

*AF = atrial fibrillation. HF = heart failure.*

### Stroke risk in relation to anticoagulation, age, and stroke risk score

Prescription of an anticoagulant was associated with reduced risk of stroke across the cohort (HR 0.65, 95% CI = 0.62 to 0.67) and this was consistent across age bands (Supplementary Table S3). However, in a post-estimation analysis, people with HF and AF (HR 1.68, 95% CI = 1.62 to 1.75) or AF alone (HR 1.76, 95% CI = 1.70 to 1.83) who were prescribed an anticoagulant remained at some increased risk of stroke compared with people with neither condition.

In comparison with people with neither HF nor AF within specific age bands, the relative rate of stroke was highest for people with HF diagnosed aged <65 years, particularly among people who were not treated with anticoagulation (Supplementary Tables S1, S4, and S5). In a second post-estimation analysis, risk of stroke in relation to age at diagnosis with HF and/or AF was compared (Supplementary Table S6). The HR for stroke was lower in people with HF alone diagnosed aged <65 years than in people with AF alone diagnosed aged ≥65 years (HR 0.28, 95% CI = 0.26 to 0.30); that is, when in comparison with the cohort with AF who would be likely to start taking an anticoagulant.

The cumulative probability of stroke was similar between people with AF alone or HF and AF when comparing within categories of CHA_2_DS_2_-VASc score (Supplementary Table S7).

### Sensitivity analyses

Similar results were found in the main Cox and Fine–Gray models in the landmark analysis from study index date and 5-year follow-up (Supplementary Tables S8 and S9). There was also no change in the summary findings in a sensitivity analysis including 5677 fatal stroke events recorded in ONS data, but not captured in CPRD or HES data (Supplementary Table S10). Of these, 4088 listed stroke as the primary cause of death.

## Discussion

### Summary

Both HF and AF were associated with an increased risk of stroke. In the Cox analysis, the relative stroke risk was similar among people with HF and AF or AF alone. However, accounting for the competing risk of death led to a significant attenuation in the relative stroke risk among people with HF and AF, but not AF alone. This reflects the high mortality among people with HF, with only a minority having a stroke before death. These findings suggest that people with HF and AF who survive for an extended period are at an increased risk of stroke, but that HF may be a relatively poor predictor of stroke among the population with AF. Conversely, younger people with HF may have most to gain from anticoagulation.

### Strengths and limitations

The analysis draws on a large primary care dataset, providing sufficient power to make comparisons between key subgroups and providing results that are generalisable to the majority of patients with HF and AF, who are managed in primary care. Data were triangulated with secondary care data to identify all key exposure and outcome variables. A competing risks analysis was conducted to explore the prognostic importance of HF and AF in the context of a high rate of mortality.^19^^,^^20^

Analyses relying on routinely collected primary care data are limited by the accuracy of clinical coding. The type of stroke was not coded in nearly half of cases and limited categorisation of HF based on ejection fraction was found. This makes it unclear how trends in the respective prevalence of HF with preserved or reduced ejection fraction and changes in treatment over time might have impacted on the results. However, ‘congestive’ HF was included in the original CHA_2_DS_2_-VASc score without qualification by ejection fraction,^3^ and the absence of these codes in the electronic health record suggests GPs are not differentiating stroke risk in HF based on ejection fraction.

The proportion of eligible patients with AF who were prescribed anticoagulation was lower than anticipated. This may reflect changes in practice over time, given that antiplatelets were recommended for stroke prevention in the early years of the study,^21^ or the relatively narrow time windows used to identify prescriptions. Using short time windows for data extraction may also underestimate the importance of time-varying covariates and miss relevant interim codes, such as changes in treatment. For example, some patients may have started anticoagulation at a follow-up date that was beyond the period when treatment data were extracted, and other patients who were initiated on anticoagulation may have subsequently stopped treatment.

It is possible that there is survivor treatment selection bias, whereby individuals who are perceived by their doctor to have a better prognosis might be more likely to be treated with anticoagulation, though the results were consistent across age categories. The authors did not control for the prescribing of other medications that improve prognosis in HF or cardiovascular disease, as this was outside the scope of this analysis. There may be residual confounding, such as with respect to differences in monitoring and treatment between people with AF alone or people with HF and AF that explain the results seen. Nonetheless, such factors would be difficult for clinicians to account for when considering the importance of HF when estimating stroke risk in people with AF, so the approach is likely to reflect that taken in practice.

Some recent methodology articles have suggested time-varying covariates should not be incorporated into competing risks models, as the model requires that the value of the time-dependent covariate be known after the competing event, even if that event is death.^11^^,^^22^ However, the landmark sensitivity analysis in the present study demonstrated broadly similar results.

### Comparison with existing literature

An observational Danish cohort study included 289 353 people with HF and 1 446 765 matched individuals and reported that people with HF were at increased risk of both ischaemic and haemorrhagic stroke at short-(adjusted rate ratio 2.08, 95% CI = 1.99 to 2.18) and long-term follow-up (adjusted rate ratio 1.54, 95% CI = 1.51 to 1.58).^23^ This association was maintained irrespective of presence of AF or anticoagulation. Clinical trials, such as CHARM-Preserved and I-Preserve, have reported that HF with preserved ejection fraction is also associated with an increased stroke risk, irrespective of the presence of AF.^24^ The present study reports a similar HR for stroke among people with HF and AF in the fully adjusted Fine–Gray model compared with the Danish study.

However, the evidence supporting the inclusion of HF as a stroke risk factor in CHA_2_DS_2_-VASc remains uncertain. In a key validation study of CHA_2_DS_2_-VASc using the EuroHeart survey dataset, the odds ratio for a thromboembolic event among people with HF was just 0.72 (95% CI = 0.27 to 1.88).^8^ A subsequent external validation in the Swedish Atrial Fibrillation cohort reported that HF was associated with an increased risk of stroke in a univariate analysis (HR 1.28, 95% CI = 1.21 to 1.35) but not multivariable analysis (HR 0.98, 0.93 to 1.03).^20^ A systematic review analysing the relative importance of stroke risk factors identified 12 studies that had assessed HF and found that only three had reported a positive association between HF and risk of stroke.^25^

Competing risks may in part explain these differing results. The present study results suggest that HF is an aetiological risk factor for stroke yet its prognostic significance is reduced by the high incidence of death. Previous research on competing risks in people with AF has demonstrated that using the inverse of the KM function overestimates the risk of stroke by 39%, particularly in those with a heavy burden of cardiovascular comorbidity.^26^ Similarly, using a Cox model for calibration of the CHA_2_DS_2_-VASc led to a significant overestimate of stroke risk compared with calibration with the Fine–Gray model.^26^

### Implications for research and practice

The authors plan to continue this research by externally validating the CHA_2_DS_2_-VASc score accounting for competing risks, which was beyond the scope of this analysis. Although many patients with HF and AF will still benefit from anticoagulation, the risks and benefits of treatment need to be carefully considered in patients known to have a poor prognosis. Optimising medical therapy for people with HF is important to improve prognosis.^27^^,^^28^

Overall, people with HF and AF were at increased risk of stroke. However, the prognosis for people with HF is often poor, and after accounting for the competing risk of death the stroke incidence among people with HF and AF was lower than anticipated. Existing approaches to stroke risk estimation using the CHA_2_DS_2_-VASc score do not account for competing risks and may overestimate the benefit of anticoagulation for patients with HF and AF who have a very poor prognosis.

## Data

This study is based in part on data from the CPRD obtained under licence from the UK Medicines and Healthcare products Regulatory Agency and cannot be shared. Equivalent data can be obtained directly from CPRD with relevant ISAC approval. The data are provided by patients and collected by the NHS as part of their care and support. The interpretation and conclusions contained in this study are those of the authors alone.
